# Collecting sexual assault history and forensic evidence from adult women in the emergency department: a retrospective study

**DOI:** 10.1186/s12913-018-3205-8

**Published:** 2018-05-29

**Authors:** Pamela Tozzo, Elena Ponzano, Gloria Spigarolo, Patrizia Nespeca, Luciana Caenazzo

**Affiliations:** Department of Molecular Medicine, Laboratory of Forensic Genetics, University of Padova, Via Falloppio 50, 35121 Padova, Italy

**Keywords:** Sexual assault, Victim’s case history, Forensic genetics, Healthcare professionals

## Abstract

**Background:**

The objective of this retrospective study was to examine the discrepancy between information derived from written medical reports and the results of forensic DNA analyses on swabs collected from the victims in 122 cases of alleged sexual assault treated at the Emergency Department of Padua Hospital. The examination of discrepant results has proved useful to support a broader application of sexual assault management, particularly during the taking of case history.

**Methods:**

The Laboratory of Forensic Genetics of Padua University have processed samples from 122 sexual assault cases over a period of 5 years.

**Results:**

Of the 103 cases in which the victim reported a penetration and ejaculation, only 67 (55% of all the samples) correlated with positive feedback match from the laboratory. In 36 cases in which the patient reported penetration with ejaculation, no male DNA was found in the samples collected. Therefore, there was a total of 41 cases in which the patient’s report were not supported by laboratory data. In the remaining ten cases, which had an ambiguous history, 3 tested positively for the presence of male DNA.

**Conclusions:**

To avoid discrepancies between the medical reporting and reconstruction of sex crimes, it is crucial to deploy strategies which focus not only on the technical aspects of evidence collection, but also on how the victim’s story is recorded; such efforts could lead to better management of sexual assault victims, and to a strengthened legal impact of forensic evidence and of crime reconstruction.

## Background

Sexual assault is a crime which is frequently encountered by healthcare professionals and forensic scientists as the World Health Organization highlights [1].

Official statistical data from the second ISTAT survey (2014) on violence against women show that 31.5% of women in Italy between the ages of 16 and 70 years are estimated to have been victims of physical or sexual violence during their lifetime: 20.2% have suffered acts of physical violence; 21% were victims of sexual violence; and 5.4% suffered more severe forms of sexual violence, such as rapes or attempted rapes [2].

Healthcare professionals treating victims of sexual assault admitted to Emergency Departments (ED) need to deal not only with clinical priorities, but also with the emotional suffering and anguish characterizing the experience of this type of patients [3]. Therefore, it is important to ensure an approach based on empathy, understanding and a willingness to listen [4, 5]. Furthermore, they can then more effectively contribute to violence management by documenting injuries, and by collecting and sampling biological evidence for forensic purposes, including those of forensic interest [6–16].

Despite the fact that indicators were published in the FIGO guidelines in 2010 [11], there is still even today a lack of consistency in how they are applied in some hospital settings. The case histories collected from the victim in the  ED are still sometimes inadequate or incomplete to determine how the case should be reconstructed [17]. Recording a victim’s history in cases of sexual assault is still problematic in Italy, which may be for a number of reasons. For example, healthcare professionals may not be adequately trained to do take this, and they often struggle with an excessive workload. The unit where the patient is admitted may not be a referral center for this type of case, so the healthcare providers involved may lack the appropriate means and may be unable to call in psychologists specially trained to handle this type of patient [18–20]. These factors necessitate a standardized medical approach to such patients as much as possible, and the adoption of an appropriate protocol to collect details and samples that could be useful for forensic purposes. It is also of paramount importance to provide specific training for healthcare professionals in this setting [8, 9].

The ED at Padua Hospital works in conjunction with the Forensic Genetics Laboratory of Padua University, which stores biological samples collected during the medical examination of victims of sexual assault. These biological samples are usually transferred to the laboratory together with a written report of the medical examination performed at the ED.

The objective of this retrospective study was to examine the discrepancy between information derived from written medical reports and the results of forensic DNA analyses on swabs collected from the victims in 122 cases of alleged sexual assault visited at the ED of Padua Hospital and to provide descriptive data on medico-legal findings. The examination of discrepant results could prove useful to provide support for a broader application of sexual assault case management, particularly in the collection of patient history.

The analyses were performed using the Christmas tree assay and the Y haplotype typing for the sole purpose of detecting the presence of any male DNA on biological samples (vaginal, rectal and vulvar swabs), since male DNA can be expected to be found if penile penetration and ejaculation have been reported. No autosomal short tandem repeat (STR) polymorphism analyses were performed to avoid any differential lysis [21], given that identifying the perpetrator’s genetic profile was not a goal of this study.

## Methods

Data were obtained from a retrospective review of the case records and forensic laboratory results obtained on biological evidence collected from the victims at the Laboratory of Forensic Genetics of the Department of Molecular Medicine, University of Padua.

All samples from 122 sexual assault cases processed by the laboratory between 1st January 2010 and 31st December 2014 were considered. In all these cases, the victims were women; they all underwent medical examination, and biological evidence was collected by means of vaginal, vulvar and rectal swabs. All the samples were originally dried and stored at − 20 °C until needed.

In particular:

- Seventy one cases were related to judicial investigations, since the biological evidence was collected as part of a technical consultation by order of the Court;
- Fifty one cases were from the hospital archives, since the biological samples were collected during a medical examination conducted at the Obstetrics and Gynecology ED of the Padua Hospital and stored at the Laboratory of Forensic Genetics; in these cases, the victims had not filed a legal complaint, and the deadline for doing so according to Italian law had already expired.

The study was divided into two main phases. The first was completed at the Forensic Genetics Laboratory and involved searching for a male genetic profile on the samples collected from patients who reported having been the victim of a sexual assault. In the second phase, the data obtained from the laboratory analyses were compared, for all 122 cases, with the written medical reports (which included the patient’s account of the episode, if any). For the 71 cases in which judicial investigations were undertaken, we were also able to compare the results of this study with the Y haplotype of the person indicated by the victim as the perpetrator.

Body swabs were tested for any presence of spermatozoa using the Christmas tree assay method [22]. DNA was extracted from swabs using the QIAampDNAMicro kit (QIAGEN Hilden, Germany) according to the manufacturer’s protocol. Standard DNA amplification was performed for each sample using the AmpFLSTRYfilerAmplification kit (Applied Biosystems, Foster City, CA, USA), which amplifies 17 Y-STRs, following the manufacturer’s recommendations. The amplified alleles were analyzed with the ABI-PRISM 3130 Genetic Analyzer (Applied Biosystems).

The laboratory findings were compared with the patients’ reports and the outcome of their physical examination, as recorded on standard evidence collection documents.

The requirement for informed patient consent and ethical committee approval were waived because of the anonymous nature of the data (i.e. it was impossible to correlate them with any individual).

## Results

Among the 103 cases in which the victim reported a penetration and ejaculation, only 67 (55% of the whole sample) coincided with a positive feedback from the laboratory, i.e. spermatic material was found, as expected, and a Y haplotype was obtained. In 6 cases, DNA typing revealed a different Y-haplotype from that of the individual indicated by the victim as the aggressor.

Among the nine cases in which the medical report was negative for penetration and ejaculation, the assays yielded valid male profiles in 5 cases (4% of the whole sample): these were cases in which the victim reported having been raped, but her description of the incident supported the assumption that there would be no semen in her vaginal canal (e.g. penetration with a condom or vaginal touching, or other forms of violence without penetration).

The remaining ten cases involved an ambiguous story in the medical record due to the victims’ inability to recollect the assault either because of alcohol or drugs intake or because of a mental condition: three of these cases resulted in positive presence of male DNA (30% of the ambiguous cases).

It is worth emphasizing that a story of sexual assault with vaginal penetration and ejaculation coincided with the discovery of male DNA using laboratory methods in more than one in every two cases (55%). Medical reports and laboratory findings were also consistent in the four cases in which the patient reported no penetration; no male DNA was found (3% of the whole sample). This led to a total of 71 cases (58% of the whole sample) in which there was a correlation between the victim’s description of the event and the forensic genetics laboratory findings. On the other hand, no correlation between reported penetration and male DNA in the samples collected from the victim was found in 36 cases (29% of the total). There was also a discrepancy in five cases (4% of the total) in which, vice versa, the medical report was negative, but male DNA was detected in the biological samples. This led to a total of 41 cases of inconsistency between the medical reports and the laboratory data (33% of the whole sample). Male DNA was only detected in cases in which the presence of spermatic material had first been identified using the Christmas tree method.

It is important to highlight that the time elapsing between the reported sexual assault and the collection of biological evidence often wasn’t stated on the written medical reports, and only in 12 cases - in the sample as a whole - this interval was reportedly less than 24 h.

**Fig. 1:**
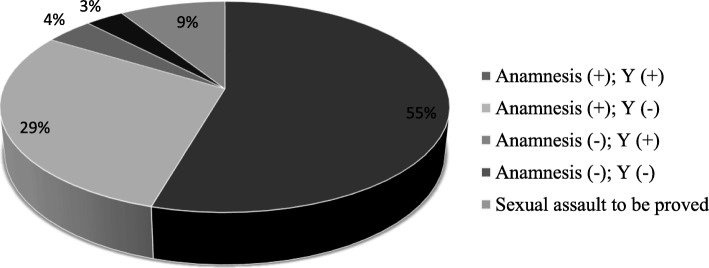
Graphic representation of the results obtained, considering the concurrence between the anamnestic data and the result obtained in the forensic genetics laboratory. Anamnesis (+): history indicative of penetration and ejaculation; History (−): history on the basis of which the expectation of finding male DNA because of the violence was null; Y (+): detection of male genetic material; Y(−):male DNA not detected

The results are summarized in Fig. 1.

## Discussion

Among the 103 cases in which the victims reported sexual contact with ejaculation, we found male biological material in 67 of their biological samples. In six of these cases, moreover, the Y haplotype differed from that of the person accused by the victim as the aggressor. In three cases, the male DNA found in the swabs collected from the victim belonged instead to the victim’s partner, with whom she had evidently had sexual intercourse prior to the assault, but had failed to mention in the report.

The time elapsing between the reported sexual assault and the collection of biological evidence was often not stated on the written medical reports as reported in the results. This is an important point to bear in mind because the discrepancy between reports of penetration and negative laboratory findings in most of the 36 cases in which this situation was identified (29% of the sample as a whole) may be due to the time elapsing between the assault and the medical examination being excessively long. It was only in seven of these cases that biological evidence was collected within 24 h after the aggression. In another six cases, the time elapsing between the assault and the collection of evidence on the victim’s body was clearly too long, and in the remaining 23 cases the interval between the assault and the medical examination was not stated in the medical records. In this regard, the protocols in current use specify that gynecological examinations should be conducted to keep the time elapsing before the collection of evidence “reasonably short”.

For the seven cases in which penetration was reported but laboratory results were negative even though the medical examination had been performed within 24 h after the assault, it may be hypothesized that either the victim or the physician were inaccurate and/or inconsistent in reporting the assault. Other potential confounders include: a health professional’s excessively brief recording of the facts, lack of detail in recording the patient’s story, obtaining vaginal swabs more than 24 h after the event and failure to record this time interval. Merely indicating “reported sexual violence” in the written medical report accompanying the samples (as it is often the case) is not enough to describe the event thoroughly, as it provides no useful information for reconstructing the dynamics of the sexual assault.

The retrospective nature of this investigation prevented any accurate assessment of other details omitted in the medical reports that might have influenced the discrepancy, such as whether victims had bathed or changed their underwear or clothes before biological evidence was collected at the ED. In Italy, only police and judicial authorities may request analyses of biological trace evidence to forensic genetics laboratories. Consequently, in assault cases not reported to the police, no such analyses could be performed.

In some cases, the detection of male DNA on samples obtained from the victim was unexpected. Possible reasons for this situation might include, for example, either a tearing during intercourse, or the presence of biological material belonging to a male other than the aggressor. We did not verify this hypothesis by means of further analyses because we had no male genetic profiles available for comparison since legal action had not been taken by the victims. In such cases, obtaining a more thorough medical report might facilitate the interpretation of the laboratory data; for example, it would be useful to ask patients if they had been involved in any consensual sexual activity shortly before or after the reported sexual assault.

Regarding the 10 unclear cases in which it was impossible to establish from the medical report whether or not sexual assault had occurred, male DNA was found in three cases (2.5% of the whole sample). However these data were not very informative because it was difficult to obtain further details about the episode, given the victims’ altered level of consciousness during the assault and when they were admitted to hospital.

This study shows a considerable discrepancy between laboratory findings and details recorded in medical reports written at the ED in the case of victims of sexual assault. The reason for this discrepancy may be twofold. On the one hand, the victim’s story might be inaccurate because of the trauma associated with sexual violence possibly producing an important impact on some victims’ ability to provide an account of events. On the other hand, medical reports may be either superficial or lack details, thus incompletely describing the information and evidence collected. The advice to healthcare professionals should be to take an investigative approach which takes into consideration the fact that victims’ recollection of events may be impaired by emotional distress, drugs, alcohol, etc., and that the patients’ recollection may change at a later stage. Similarly, patients may have been subjected to acts that they were not aware of.

For the reasons above our findings suggest that hospital services which deal with victims of sexual assault should pay more attention to the methods used to obtain and record the material collected during the victim’s medical examination, and to interview patients about the episode. The paucity of publications on studies aiming to improve the way in which medical reports are drawn up on victims of sexual violence also confirms, albeit directly, the shortcoming in this field of research.

Furthermore, the hypothesis that discrepancies between medical reports and laboratory findings can be attributed to victims giving untrue accounts of their experience at the ED, is closely correlated to how such patients are managed. Victims of sexual assault have often suffered a major trauma and a far from negligible emotional stress, so it may be that during the interview with the ED staff they fail to mention or alter important details concerning the aggression, either because they are ashamed or because they forget.

To avoid discrepancies between the medical reporting and the reconstruction of sex crimes, it is crucial to use strategies which focus not only on technical aspects of evidence collection, but also on the way the victim’s story shall be recorded. Such efforts could lead to better management of sexual assault victims, and to securing their legal rights. Within this setting the involvement of healthcare professionals specialized in the forensic field is paramount both for conducting accurate clinical examination for collecting a detailed documentation of physical injuries and for sampling biological evidence for forensic purposes. Rigorous documentation of evidence is a crucial requirement for any healthcare professional working in this area, and this would be beneficial to sexual assault victims as much as to healthcare professionals working in this context. A recent study highlighted that failure to adequately assess patients reporting sexual violence was reported as a common complaint made against healthcare professionals working in this area [23].

The examination of the victim, which is performed by the ED clinician and the forensic doctor, should be completed as soon as possible after the assault to avoid the loss of important trace evidence, and to strengthen the legal impact of forensic evidence and of crime reconstruction.

In all cases, appropriate professional training is required to ensure that medical examiners are competent in the medical reporting of sexual assault cases, and also for the purpose of documenting the evidence of a sexual assault. ED can do an excellent job for managing the clinical needs of sexual assault victims, and can also maximize the chances for female victims engaging the necessary health service. From a forensic viewpoint, obtaining an accurate description of the episode and understanding of its dynamics can also be crucial as medical documentation may be used in courts.

## Conclusion

The results show a discrepancy between the case history and the biological evidence obtained. These differences could be reduced through various strategies. First of all, it is desirable that the FIGO guidelines [11] are deployed consistently. Thus, focus should not only be on the technical aspects of evidence collection but mostly on the accurate record of the victim’s story and on the way detailed reports may improve the reconstruction of the assault.

Secondly, the presence of health care professionals specialized in the forensic field in ED would be desirable for supporting the personnel involved in the assistance to the victims and for enabling the collection of evidence. In particular, this would be relevant for initial history taking, inspection of the victim and collection of biological samples for forensic analysis.

Furthermore, systematic training courses for healthcare professionals should also cover the need to obtain and analyze forensic evidence as well as medical reports, which together account for a substantial part of the evidence presented in court.

Improving the methods for collecting patients’ stories and conducting medical examination, as well as a sensitization of healthcare staff towards patients’ physical and emotional needs would enable more detailed information to be obtained from these patients, even when they are uncooperative.

In order to understand the dynamics of the episode in rape cases, and to avoid discrepancies between medical reports and legal reconstructions of sexual crimes, it is crucial to provide victims with support, and to maximize their confidence in the healthcare providers who first attend to them, usually at the ED.

## Abbreviation

ED: Emergency Department
